# *Pseudomonas atagosis* sp. nov., and *Pseudomonas akappagea* sp. nov., New Soil Bacteria Isolated from Samples on the Volcanic Island Izu Oshima, Tokyo

**DOI:** 10.1007/s00284-020-01943-2

**Published:** 2020-03-18

**Authors:** Yuh Morimoto, Kazuki Uwabe, Mari Tohya, Keiichi Hiramatsu, Teruo Kirikae, Tadashi Baba

**Affiliations:** 1 Center of Excellence for Infection Control Science, Graduate School of Medicine, Juntendo University, 2-1-1 Hongo, Bunkyo-ku, Tokyo, 113-8421 Japan; 2 School of Medicine, Juntendo University, 2-1-1 Hongo, Bunkyo-ku, Tokyo, 113-8421 Japan; 3 Department of Microbiology, Faculty of Medicine, Juntendo University, 2-1-1 Hongo, Bunkyo-ku, Tokyo, 113-8421 Japan

## Abstract

During the exploration of microbial natural resources, two strains of *Pseudomonas*, PS14^T^ and PS24^T^, were isolated from samples taken from Izu Oshima, a volcanic island located 120 km southwest of central Tokyo. Phylogenetic analysis based on 16S rRNA gene sequences showed that PS14^T^ was most similar to *Pseudomonas baetica* a390^T^ (99.6%) and *Pseudomonas helmanticensis* OHA11^T^ (99.5%), and that PS24^T^ was most similar to *Pseudomonas qingdaonensis* JJ3^T^ (98.8%) and *Pseudomonas lutea* OK2^T^ (98.7%). The major fatty acids of these two strains were C_16:0_ and C_17:0_ cyclo, summed feature 3 (C_16:1_ ω6c and/or C_16:1_ ω7c), and summed feature 8 (C_18:1_ ω7c and/or _18:1_ ω6c). The phylogenetic analyses, DNA-DNA hybridization results and phenotypic traits indicated that PS14^T^ and PS24^T^ constitute two novel species, *Pseudomonas atagosis* sp. nov. (type strain PS14^T^ = CECT 9940^T^, = LMG 31496^T^) and *Pseudomonas akappagea* sp. nov. (type strain PS24^T^ = CECT 9941^T^, = LMG 31497^T^), respectively. The sequence data of the draft genomes of PS14^T^ and PS24^T^ were deposited in the GenBank database under accession numbers VXCA00000000 and VXCP00000000, respectively, and the sequence data of their 16S rRNA genes were deposited in the GenBank database under accession numbers MN396717 and MN382268, respectively.

**Electronic supplementary material:**

The online version of this article (10.1007/s00284-020-01943-2) contains supplementary material, which is available to authorized users.

## Introduction

The genus *Pseudomonas* was first described at the end of the nineteenth century [1]. *Pseudomonas* strains are Gram-negative, rod-shaped, motile, catalase-positive and oxidase-positive bacterial cells. These bacteria have been isolated from various environments worldwide, including soil, animals, plants, and water [2]. To date, the List of Prokaryotic Names with Standing in Nomenclature (https://www.bacterio.net) includes 255 species of *Pseudomonas*, including 18 subspecies.

During the exploration of microbial natural resources, we collected soil samples from Izu Oshima, in January 2017. PS14^T^ was isolated from soil collected at Mt. Atago, which is located in the northwest part of the island. Mt. Atago, where *Castanopsis sieboldii* trees grow, is the transitional final stage, known as a climax community. PS24^T^ was isolated from a red scoria cone located on the west coast of the island, which the local inhabitants call Akappage. The present study describes the phenotypic and phylogenic characteristics of these two strains. These characteristics indicate that these two strains represent novel species of the genus *Pseudomonas*.

## Materials and Methods

### Strains and Growth Conditions

Approximately 5 g of the ground surface was collected at eight locations on Izu Oshima, including Mt. Atago (34° 77′ 06" N, 139° 35′ 98" E) and Akappage (34° 77′ 50" N, 139° 34′ 99" E). Approximately 0.5 g of soil samples were suspended in 5 ml of 0.9% NaCl solution, and 0.1 ml of the suspension of each sample was spread onto *Pseudomonas* spp. selective medium (Pseudomonas CFC/CN agar, Merck). The plates were incubated for 48 h at room temperature. 20 and 76 colonies appeared from the Mt. Atago and Akapage samples, and several colonies with different colony morphologies were selected and purified with a single colony isolation. PS14^T^ and PS24^T^ were two of the selected isolates. The reference strains *Pseudomonas helmanticensis* LMG 28168^T^, *Pseudomonas lutea* LMG 21974^T^, *Pseudomonas rhizosphaerae* LMG 21640^T^ and *Pseudomonas bohemica* LMG 30182^T^ were obtained from Belgian Coordinated Collections Microbiology (BCCM). *Pseudomonas koreensis* JCM 14769^T^ and *Pseudomonas qingdaonensis* JCM 32579^T^ were provided by the RIKEN BRC through the National BioResource Project of the MEXT/AMED, Japan. *Pseudomonas helmanticensis* LMG 28168^T^ and *Pseudomonas bohemica* LMG 30182^T^ were imported under the permit of the Minister of Agriculture, Forestry and Fisheries, Japan, in accordance with the Plant Protection Law. All these strains were cultured in tryptic soy broth (TSB, Becton Dickinson).

### Morphological, Physiological and Biochemical Studies

Cell morphology was examined by scanning electron microscopy (Hitachi S-4800). Colony morphology was assessed on tryptic soy agar (TSA, Becton Dickinson) plates after culture for 24 h at 28 °C. Growth at various temperatures was tested by culturing in Luria–Bertani broth (LB, Becton Dickinson) [3]. Briefly, overnight cultures of tested strains were adjusted to OD_600nm_ = 0.225, and 20 μl of each sample were inoculated into 10 ml of LB. These strains were incubated at 5, 8, 12, 24, 28, 32, 36 and 40 °C while shaking at 25 rpm in a photorecording incubator (TN-2612; ADVANTEC, Tokyo, Japan). Gram-staining was performed by a staining kit (Muto Pure Chemicals co. ltd, Tokyo Japan). Motility was directly assessed using a Bacteria Self-Checker mil-kin® (https://www.mil-kin.com/). Fluorescent pigmentation was assessed on King B medium (Eiken, Tokyo Japan), as described previously [4]. Oxidase activity as assessed using Cytochrome Oxidase Test Strips (Nissui, Tokyo, Japan). Catalase activity was analyzed by dropping 3% hydrogen peroxide solution onto the cells and monitoring the production of bubbles. Growth at different NaCl concentrations was assessed in nutrient broth (Becton Dickinson) [3] containing 0, 1, 2, 3, 4, 5, 6 and 7% NaCl. Growth at different pH levels (5, 6, 7, 8, 9, and 10) was investigated by adding hydrochloric acid or sodium hydroxide to 7.5 ml of twofold-higher TSB and 3 ml of buffer agent (MOPS for pH 5 to pH 7, HEPES for pH 8 to pH 9, and CAPS for pH 9 to pH 10). The broth was diluted with sterile water to adjust the TSB concentration to onefold. API 20 NE strips (bioMérieux) and Biolog GN3 MicroPlates were used according to the manufacturers’ instructions. API 20 NE and GN3 tests for *Pseudomonas granadensis* DSM 28040^T^ was performed by German Collection of Microorganisms and Cell Cultures GmbH (DSMZ).

### Chemotaxonomic Characterization

Fatty acid methyl ester analysis was performed at Techno Suruga Laboratory Co., Ltd (Shizuoka, Japan). Fatty acids were prepared as described by MIDI Microbial Identification System [5] and analyzed using the Sherlock Microbial Identification (MIDI) system (version 6.0).

### Genomic DNA Preparation, Sequencing, and Assembly

Genomic DNA was extracted from PS14^T^ and PS24^T^ using QIAamp DNA Mini Kits (Qiagen), and genomic libraries of both strains were prepared using Nextera XT DNA Library Preparation Kits (Illumina). Paired-end sequencing was performed using MiSeq Reagent Kits v3 (600-cycles) through the Illumina MiSeq platform. De novo assembly was performed using CLC Genomics Workbench v7 (Qiagen). The DNA sequences of the 16S rRNA genes were analyzed using BigDye® Terminator v3.1 Cycle Sequencing Kits and an ABI PRISM 3100 Genetic Analyzer (Applied Biosystems, Life Technologies, Carlsbad, CA), along with the primers 8F (5′-AGAGTTTGATCCTGGCTCAG-3′) and 1541R (5′-AAGGAGGTGATCCAGCCGCA-3′) [6].

### Phylogenetic Analysis

Sequences were aligned using CLUSTAL W software and phylogenetic trees were constructed using MEGA 7.0 software [7]. Evolutionary distances were calculated using Tamura’s 3-parameter model [8]. To account for heterogeneity of substitution rate among nucleotide sites, the discrete gamma model with 5 categories was used. Phylogenetic trees were reconstructed using maximum-likelihood (ML) methods [9]. The sequences of all *Pseudomonas* type strains used for the analysis except *Pseudomonas helmanticensis* LMG 28168^T^ were retrieved from the National Center for Biotechnology Information (NCBI) GenBank database and EzBioCloud (https://www.ezbiocloud.net/). *Pseudomonas helmanticensis* LMG 28168^T^ (GOLD ID Gp0112928) was retrieved from Department of Energy Joint Genome Institute (https://www.jgi.doe.gov) under Genomes Online Database IMG.

### Genome Analysis

The similarity of the sequenced genomes to genomes of other type strains was determined based on the Average Nucleotide Identity with OrthoANIu algorithm [10] and Genome-to-Genome-Distance (GGDC) version 2.1 software [11]. The GGDC results were based on formula 2, which is independent of genome length and is therefore recommended to use for incomplete draft genomes.

## Results and Discussion

**Fig. 1:**
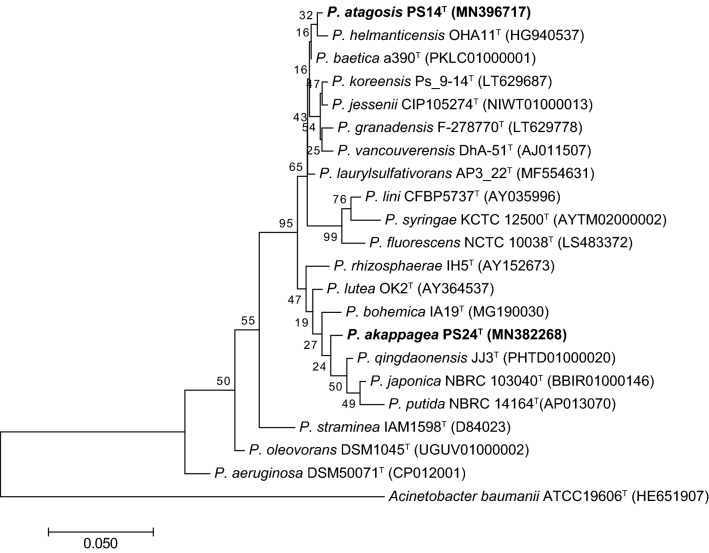
Maximum-likelihood (ML) tree based on 16S rRNA gene sequences (1459 bp) showing the relationships of the strains, *P. atagosis* sp. nov. PS14^T^ and *P. akappagea* sp. nov. PS24^T^, with related type strains of the genus *Pseudomonas*. The ML tree was reconstructed using Tamura's 3-parameter model +G. The discrete gamma model with 5 categories were used. Bootstrap values, expressed as percentages of 1000 replications, are shown at the branching points. GenBank accession numbers are given in parentheses and in Table S1

Phylogenetic trees were constructed based on the 16S rRNA sequences (1459 bp) of PS14^T^ and PS24^T^ and of representative *Pseudomonas* strains (Fig. 1). GenBank accession numbers are listed in Table S1. The highest interspecific sequence similarities that were found between strain PS14^T^ and its phylogenetic neighbors were *Pseudomonas baetica* a390^T^ (99.6%) and *P. helmanticensis* OHA11^T^ (99.5%), and that of PS24^T^ were *P. qingdaonensis* JJ3^T^ (98.8%) and *P. lutea* OK2^T^ (98.7%).

**Fig. 2:**
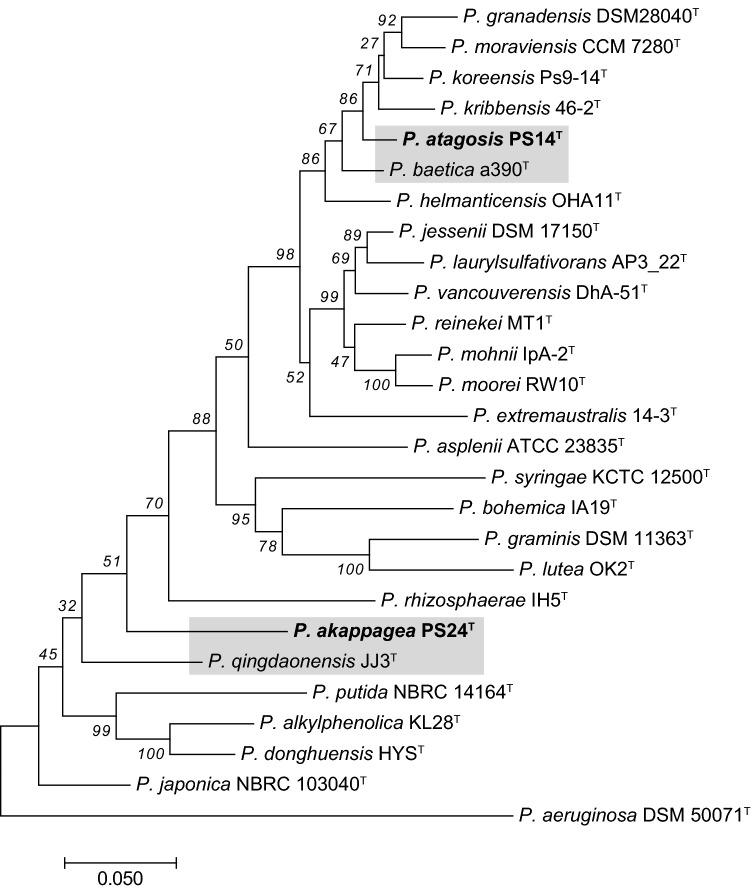
Maximum-likelihood (ML) tree based on the sequences of the housekeeping genes, 16S rRNA, *gyrB*, *rpoD* and *rpoB*, showing the relationships of the strains, *P. atagosis* sp. nov. PS14^T^ and *P. akappagea* sp. nov. PS24^T^, with related *Pseudomonas* type strains. The ML tree was reconstructed using Tamura's 3-parameter model +G. The discrete gamma model with 5 categories were used. Bootstrap values, expressed as percentages of 1000 replications, are shown at the branching points. Grey boxes indicate that the strains of this study and the closest type strain based on 16S rRNA comparison. The accession numbers of each sequence are listed in Tables S1 and S2

Figure 2 is a phylogenetic tree constructed based on concatenated sequences of 16S rRNA and three housekeeping genes linked in the order 16S rRNA (1459 bp)–*gyrB* (801 bp)–*rpoD* (718 bp) –*rpoB* (915 bp) (Fig. 2). These sequences were retrieved from the genome sequences, and GenBank accession numbers of these genes are listed in Tables S1 and S2. Strain PS14^T^ clusters in a separate branch that is related to a group including *P. baetica*, *P. helmanticensis* and *P. koreensis*. PS24^T^ was placed near *P. qingdaonensis* and *P. rhizosphaerae*. These results indicate that both of these Izu Oshima strains belong to the *P. fluorescens* lineage, but they are distinct from other species in that lineage.

### Genomic Analysis

The DNA G+C contents of PS14^T^ and PS24^T^ were found to be 59.6% and 60.2%, respectively. Assessments of ANI scores and dDDH values of PS14^T^, PS24^T^ and closely related strains are listed in Table S3. The highest correlations were between PS14^T^ and *P. helmanticensis*, with an ANI score of 88.3% and a dDDH score of 35.7%, and between PS24^T^ and *P. qingdaonensis*, with an ANI score of 80.8% and a dDDH score of 24.5%. These ANI and dDDH scores were lower than the cutoff values for species delineation (> 95% for ANI and > 70% for dDDH) [12], indicating that PS14^T^ and PS24^T^ are likely novel species of the genus *Pseudomonas*.

### Chemotaxonomic Characterization

The major fatty acids detected in the Izu Oshima strains were found to be C_16:0_, C_17:0 cyclo_, summed feature 3 (C_16:1_ ω6c and/or C_16:1_ ω7c), and summed feature 8 (C_18:1_ ω7c and/or _18:1_ ω6c) (Table 1). This profile is similar to that of related strains. Both PS14^T^ and PS24^T^ possess three fatty acids generally detected in the genus *Pseudomonas*, namely C_10: 0_ 3-OH, C_12: 0_ and C_12: 0_ 3-OH [11].

**Table 1:** Cellular fatty acid content of PS14^T^, PS24^T^ and closely related strains

	1	2	3	4	5	6	7	8	9	10
C_12:0_ 2OH	5.3	2.8	4.9	6.4	4.7	6.2	2.9	2.8	4.0	9.0
C_12:0_ 3OH	4.5	3.5	2.9	4.0	2.5	5.1	4.2	3.5	3.9	3.4
C_10:0_ 3OH	3.2	3.3	2.4	3.6	3.2	4.2	2.2	1.5	4.3	8.4
C_12:00_	1.6	4.3	2.0	2.1	1.5	5.0	5.7	4.9	6.2	3.0
C_16:00_	32.8	29.1	31.9	30.0	31.9	20.0	19.3	25.6	30.0	27.5
C_17:0_ cyclo	11.5	7.0	5.1	2.0	6.9	1.5	ND	1.7	8.6	8.2
C_18:00_	TR	TR	TR	TR	TR	TR	1.7	ND	TR	TR
Summed feature 3^a^	27.2	32.6	32.9	42.4	35.6	41.9	35.4	37.2	33.4	25.8
Summed feature 8^b^	10.7	15.3	15.6	8.5	12.4	13.4	27.8	21.2	7.4	11.4

Strains were cultured on TSA at 28 °C for 24 h

1, PS14^T^; 2, PS24^T^; 3, *P. helmanticensis* OHA11^T^; 4, *P. baetica* a390^T^; 5, *P. granadensis* DSM 28040^T^; 6, *P. koreensis* 9-14^T^; 7, *P. rhizosphaerae* IH5^T^; 8, *P. lutea* OK2^T^; 9, *P. bohemica* IA^T^; 10, *P. qingdaonensis* JJ3^T^. Data from taxa 3 to 5 are from reference [13], and taxa 7 and 8 are from reference [14] using the same conditions. Fatty acids (> 1% of total fatty acids) are shown

*ND* not detected, *TR* trace (Fatty acid amount < 1%)

^a^C_16:1_ ω6c and/or C_16:1_ ω 7c and/or C_15.0_ ISO 2-OH

^b^C_18:1_ ω7c and/or _18:1_ ω 6c

### Growth Conditions, Physiology, Morphology, and Biochemical Characteristics

The phenotypic features of PS14^T^ and PS24^T^ are presented in Table 2. The phenotypic features of PS14^T^ were similar to those of *P. koreensis*, although they differed in utilization of gelatin hydrolysis, d-fucose, d-arabitol, l-histidine, glucuronamide, and α-keto-glutaric acid. PS24^T^ was found to be more restricted than PS14^T^, with d-glucose being the only sugar source found to be utilized by PS24^T^.

**Table 2:** Phenotypic characteristics that differentiate the strains PS14^T^ and PS24^T^ from the closely related type strains

	1	2	3	4	5	6	8	9	7	10
GC content (%)	59.6	60.2	59.2	58.8	60.2	60.5	62.0	60.2	59.5	64.2
Fluorescence	+	−	+	+	−	+	−	−	−	+
Activity of enzymes (API 20 NE test)										
Potassium nitrate	−	+	−	−	−	−	−	−	−	−
l-arginine	+	−	−	+	−	+	−	−	−	+
Gelatin (bovine origin)	+	−	−	+	+	−	−	−	−	−
Growth on (API 20 NE test)										
d-mannose	w	−	+	+	+	+	+	+	−	−
d-mannitol	+	−	+	+	+	+	+	+	+	−
*N*-acetyl-glucosamine	+	−	+	+	+	+	−	−	−	−
Phenylacetic acid	−	+	−	−	−	−	−	−	−	+
Carbon sources (Biolog GN3)										
d-galactose	+	−	+	+	w	+	+	w	+	−
d-fucose	w	−	+	+	w	−	−	−	w	w
Inosine	w	−	w	+	−	−	−	−	w	w
d-arabitol	−	−	+	+	−	−	−	−	+	−
d-fructose-6-PO4	−	−	w	+	w	−	−	−	w	w
d-aspartic acid	−	−	−	−	−	−	−	−	−	−
d-serine	−	+	−	+	w	−	−	−	−	+
Glycyl-L-proline	−	−	−	−	−	−	−	−	−	−
Pectin	−	−	−	+	−	−	−	−	−	−
d-galacturonic acid	−	−	−	−	w	−	−	+	+	−
l-galactonic acid lactone	−	−	−	−	−	−	−	+	+	−
d-glucuronic acid	−	−	+	−	w	−	−	+	+	−
Glucuronamide	+	−	+	−	w	−	−	w	+	w
Mucic acid	+	+	+	−	w	+	−	w	+	−
Quinic acid	+	+	+	+	+	+	+	−	−	+
d-saccharic acid	+	−	+	+	w	w	−	−	+	−
p-hydroxy-phenylacetic acid	−	−	−	−	−	−	−	−	−	+
d-malic acid	−	−	w	−	−	−	−	−	−	+
Bromo-succinic acid	−	+	w	+	−	−	−	−	w	−
Tween 40	−	−	w	+	w	−	−	−	−	−
α-keto-butyric acid	−	−	−	−	−	−	−	−	−	−
Acetoacetic acid	−	−	−	−	−	−	−	−	+	−

1, PS14^T^; 2, PS24^T^; 3, *P. helmanticensis* OHA11^T^; 4, *P. baetica* a390^T^; 5, *P. granadensis* DSM 28040^T^; 6, *P. koreensis* 9-14^T^; 7, *P. rhizosphaerae* IH5^T^; 8, *P. lutea* OK2^T^; 9, *P. bohemica* IA^T^; 10, *P. qingdaonensis* JJ3^T^. All data were obtained in this study, except taxon 4, which were from reference [15] and for fluorescent data of taxon 5, which were from reference [13]

+ positive, − negative, *w* weakly positive (GN3, extremely faint color, or with small purple flecks or clumps)

### Description of *Pseudomonas atagosis* sp. nov.

This strain, which has been named for Atago Mountain, the source of the original sample, was found to be Gram-negative, motile, rod-shaped, oxidase-positive, and catalase-positive. Cells were observed to be 1.0–1.7 μm long and 0.4–0.6 μm wide. Colonies grown on TSA agar for 24 h at 28 °C were moist and creamy-white in color due to extracellular substances. Concentrated cell pellet was beige-colored. Growth was observed at temperatures of 5–32 °C, with optimum growth at 24–28 °C. The strain could grow in the presence of 0–4% (w/v) NaCl and at pH between 5 and 8 and produced a fluorescent pigment when grown on King B agar. Major fatty acids were C_16:0_, C_17:0_ cyclo, summed feature 3 (C_16:1_ ω6c and/or C_16:1_ ω7c), and summed feature 8 (C_18:1_ ω7c and/or _18:1_ ω6c). On API 20 NE tests, this strain was positive for L-arginine, gelatin, d-glucose, l-arabinose, d-mannose, d-mannitol, *N*-acetyl-glucosamine, potassium gluconate, capric acid, malic acid, and trisodium citrate. Biolog GEN III Microplate assays showed that these bacteria can utilize α- d-glucose, d-mannose, d-fructose, d-galactose, d-fucose, inosine, d-mannitol, glycerol, l-alanine, l-arginine, l-aspartic acid, l-glutamic acid, l-pyroglutamic acid, l-serine, d-gluconic acid, glucuronamide, mucic acid, quinic acid, d-saccharic acid, l-lactic acid, citric acid, *α*-keto-glutaric acid, l-malic acid, *γ*-amino-butryric acid, *β*-hydroxy-D L-butyric acid, propionic acid, and acetic acid and was able to grow in the presence of 1% sodium lactate, fusidic acid, d-serine, troleandomycin, rifamycin SV, lincomycin, guanidine HCl, niaproof 4, vancomycin, tetrazolium violet, tetrazolium blue, nalidixic acid, lithium chloride, potassium tellurite, and aztreonam. The G+C content of the type strain is 59.58%. The type strain, PS14^T^ (= CECT 9940^T^, = LMG 31496^T^), was isolated from soil collected at Mt. Atago, which is located in the northwest part of Izu Oshima, Tokyo, Japan.

### Description of *Pseudomonas akappagea* sp. nov.

This strain, which has been named for the source of the original sample, the coastal area of Izu Oshima island, called “Akappage” by the local inhabitants, was found to be Gram-negative, motile, rod-shaped, oxidase-positive, and catalase-positive. The cells were 1.25–2.0 μm long and 0.5–0.7 μm wide. Colonies grown on TSA agar for 24 h at 28 °C were beige in color. Growth was observed at 5–36 °C, with optimum growth at 24–28 °C. These bacteria could grow in the presence of 0–3% (w/v) NaCl and at pH between 5 and 8 but did not produce a fluorescent pigment when grown on King B agar. Major fatty acids were C_16:00_, C_17:0_ cyclo, summed feature 3 (C_16:1_ ω6c and/or C_16:1_ ω7c), and summed feature 8 (C_18:1_ ω7c and/or _18:1_ ω6c). On API 20NE tests, this strain was positive for potassium nitrate, d-glucose, potassium gluconate, capric acid, adipic acid, malic acid, trisodium citrate, and phenylacetic acid. Biolog GEN III Microplate assays showed that these bacteria can utilize *α*-D-glucose, glycerol, d-serine, l-alanine, l-arginine, l-aspartic acid, l-glutamic acid, l-histidine, l-pyroglutamic acid, l-serine, d-gluconic acid, mucic acid, quinic acid, methyl pyruvate, l-lactic acid, citric acid, *α*-keto-glutaric acid, l-malic acid, bromo-succinic acid, *γ*-amino-butryric acid, *β*-hydroxy-D L-butyric acid, propionic acid, acetic acid, and formic acid and was able to grow in the presence of 1% sodium lactate, d-serine, troleandomycin, rifamycin SV, lincomycin, guanidine HCl, niaproof 4, vancomycin, tetrazolium violet, tetrazolium blue, potassium tellurite, and aztreonam. The G+C content of the type strain is 60.2%. The type strain, PS24^T^ (= CECT 9941^T^, = LMG 31497^T^), was isolated from located on the west coast of Izu Oshima, Tokyo Japan.

## Electronic supplementary material

Below is the link to the electronic supplementary material.

Supplementary file1 (XLSX 11 kb) Table S1 Accession numbers used in the 16S rRNA phylogenetic analysis (Fig.1) and phylogenetic analysis based on concatenated gene sequences (Fig.2).

Supplementary file2 (XLSX 12 kb) Table S2 Accession numbers of *gyrB*, *rpoD* and *rpoB* gene sequences used in the phylogenetic analysis based on housekeeping genes.

Supplementary file3 (XLSX 18 kb) Table S3 ANI and dDDH values in comparisons of *P. atagosis* sp. nov. and *P. akappagea* sp. nov. with related type strains.
